# Assessment of the Mutational Status of NSCLC Using Hypermetabolic Circulating Tumor Cells

**DOI:** 10.3390/cancers10080270

**Published:** 2018-08-14

**Authors:** Matteo Turetta, Michela Bulfoni, Giulia Brisotto, Gianpiero Fasola, Andrea Zanello, Eva Biscontin, Laura Mariuzzi, Agostino Steffan, Carla Di Loreto, Daniela Cesselli, Fabio Del Ben

**Affiliations:** 1 Department of Medicine, University of Udine, P.le Kolbe 4, 33100 Udine, Italy; michela.bulfoni@uniud.it (M.B.); zanello.andrea@spes.uniud.it (A.Z.); laura.mariuzzi@uniud.it (L.M.); carla.diloreto@uniud.it (C.D.L.); daniela.cesselli@uniud.it (D.C.); 2 Immunopathology and Cancer Biomarkers, C.R.O. Aviano National Cancer Institute IRCCS, via F. Gallini 2, 33081 Aviano (PN), Italy; gbrisotto@cro.it (G.B.); eva.biscontin@gmail.com (E.B.); asteffan@cro.it (A.S.); 3 IOV-IRCCS, Immunology and Molecular Oncology Unit, V. Gattamelata 64, 35128 Padova, Italy; 4 DISCOG, University of Padova, V. Giustiniani 2, 35128 Padova, Italy; 5 Udine Academic Hospital, P.le Santa Maria della Misericordia 15, 33100 Udine, Italy; gianpiero.fasola@asuiud.sanita.fvg.it

**Keywords:** CTC, metabolism, glucose uptake, non-small cell lung cancer, liquid biopsy

## Abstract

Molecular characterization is currently a key step in NSCLC therapy selection. Circulating tumor cells (CTC) are excellent candidates for downstream analysis, but technology is still lagging behind. In this work, we show that the mutational status of NSCLC can be assessed on hypermetabolic CTC, detected by their increased glucose uptake. We validated the method in 30 Stage IV NSCLC patients: peripheral blood samples were incubated with a fluorescent glucose analog (2-NBDG) and analyzed by flow cytometry. Cells with the highest glucose uptake were sorted out. EGFR and KRAS mutations were detected by ddPCR. In sorted cells, mutated DNA was found in 85% of patients, finding an exact match with primary tumor in 70% of cases. Interestingly, in two patients multiple KRAS mutations were detected. Two patients displayed different mutations with respect to the primary tumor, and in two out of the four patients with a wild type primary tumor, new mutations were highlighted: EGFR p.746_750del and KRAS p.G12V. Hypermetabolic CTC can be enriched without the need of dedicated equipment and their mutational status can successfully be assessed by ddPCR. Finally, the finding of new mutations supports the possibility of probing tumor heterogeneity.

## 1. Introduction

Cancer is a leading cause of morbidity and mortality worldwide [1]. In the last years, advances in drug development enabled the employment of targeted therapies using drugs inhibiting specific pathways of critical importance for the survival of cancer cells. The growing number of treatment options, their effectiveness in a limited subset of patients, and their elevated costs, pressed the need to predict the individual response to therapy before starting the treatment. The so-called “predictive biomarkers” are therefore of critical importance in assisting clinicians in the selection of the most effective therapy. Non-Small Cell Lung Cancer (NSCLC) is one of the most prevalent and lethal cancer types [2]. Recently, multiple targeted treatments were made available for NSCLC, targeting EGFR, ALK, or BRAF mutations [2]. Current clinical practice includes the characterization of molecular subtypes of NSCLC, performed on biopsy specimens, obtained with a core biopsy. Recent advances made it possible to identify some mutations (e.g., EGFR mutations) in circulating tumor DNA, obtained from peripheral blood [3]. The sampling of tumor material from body fluids (mainly peripheral blood, but also urine, cerebrospinal fluid, tears, and saliva) is commonly addressed as a “liquid biopsy”, and it targets circulating tumor cells (CTC), circulating tumor DNA (ctDNA) or RNA, and exosomes [4]. Being minimally invasive, a liquid biopsy can be performed multiple times during the course of treatment, providing timely feedback on disease evolution, including the appearance of drug-resistances, thus enabling a rapid adjustment of therapy [4]. The quantification of CTC number and ctDNA level in the peripheral blood already proved to be prognostic in other cancer types, predicting disease progression several months in advance with respect to imaging [5,6,7,8], and providing useful information to guide targeted therapy [9,10]. Beside mere enumeration, CTC can be isolated and molecularly characterized to retrieve information on their mutational status, driving decision in targeted therapy selection, as recently proven in metastatic castration-resistant prostate cancer [9].

However, while some ctDNA technologies already reached FDA-approval to guide therapy, CTC technology is still lagging behind. CTC are cancer cells present in the peripheral blood of most solid tumors. They are rare (typically 1–100/mL), highly heterogeneous cells, and there is no clear consensus on their definition [11]. FDA has approved the quantification of CTC detected by CellSearch as a prognostic test for breast, colorectal, and prostate metastatic cancer. This test recognizes CTC as nucleated DAPI (4′,6-diamidino-2-phenylindole)(+), EpCAM (Epithelial Cell Adhesion Molecule)(+), CK (cytokeratin)(+), CD45(−) cells. However, such definition leads, in the case of NSCLC, to an insufficient sensitivity in CTC detection (approximately 20%) [12]. Other CTC subsets include CK(−) cells, cells undergoing Epithelial to Mesenchymal transition (EMT), apoptotic CTC; moreover, although there are size-based methods enriching in CTC, evidences showed the existence of CTC with a size comparable to that of white blood cells [13,14]. Each subset has been exploited by a different innovative device, with the main downside of an a priori selection of a subtype of CTC. EpCAM-based and size-based technologies are the most widespread. The restriction of validated CTC detection methods to a limited number of solid tumors is penalizing patients with a highly prevalent disease such as NSCLC, in which the antibody cocktail employed by CellSearch and other techniques are insufficient to detect CTC, as demonstrated by the low sensitivity of CellSearch in NSCLC (approximately 20%) [12] and the absence of NSCLC CTC-specific markers. This most likely happens because in such disease most CTC undergo EMT, therefore losing EpCAM expression, which is mandatory for capturing CTC with the CellSearch system [15]. Other techniques relying, for example, on an EGFR-, HER2-, and EpCAM-dependent enrichment achieved a higher, but still low sensitivity (29%), slightly increased (42%) in patients harboring tumors with altered EGFR genotype [16]. CTC characterization in NSCLC were found to express genes involved in therapy resistance such as HER3 and MET, highlighting the clinical relevance of such analysis for early detection of drug resistance in therapy monitoring [17]. A different approach, proposed by Dorsey et al., was based on the evaluation of telomerase activity, which is a specific marker of cancer cells in both the epithelial and EMT phenotypes; this study demonstrated the correlation between CTC burden and treatment success [18]. Another work, regarding EpCAM(−) detection of CTC in NSCLC, proposed a pre-enrichment step using hematopoietic cell depletion, followed by a multiparametric fluorescence analysis, comprising pan-cytokeratin, EpCAM, *N*-cadherin (specific of mesenchymal cells), and CD133 (stem-like marker). The presence of mesenchymal and stem-like cells was associated with a reduced response to platinum therapy [19]. In addition to changes in protein expression and morphology, CTC present functional alterations, summarized in a recent list of hallmarks of cancer, including an altered metabolism, that have been exploited for their detection [20,21,22,23]. One of the most well described metabolic alterations is increased glucose uptake in cancer cells, involving the upregulation of GLUT channels [20,24]. This alteration is early-appearing in cancer evolution [25], well-conserved during invasive and metastatic transformation [26], associated with aggressive phenotypes and worse prognosis [27,28,29]. Such increased glucose metabolism is notoriously exploited in the clinical routine by imaging solid tumors with positron-emission tomography [30,31]. The ability to detect both epithelial and mesenchymal phenotypes exploiting their increased uptake of glucose was recently shown by our group in a work on cancer cell lines [32], in which we also hypothesized that such method could have been applied to improve detection of CTC. Here, we report the proof-of-concept of such hypothesis by detecting and harvesting hypermetabolic CTC in the peripheral blood of NSCLC patients.

## 2. Results

### 2.1. Feasibility Study

#### 2.1.1. Tumor Cells Are Characterized by an Increased Glucose Uptake

**Figure 1:**
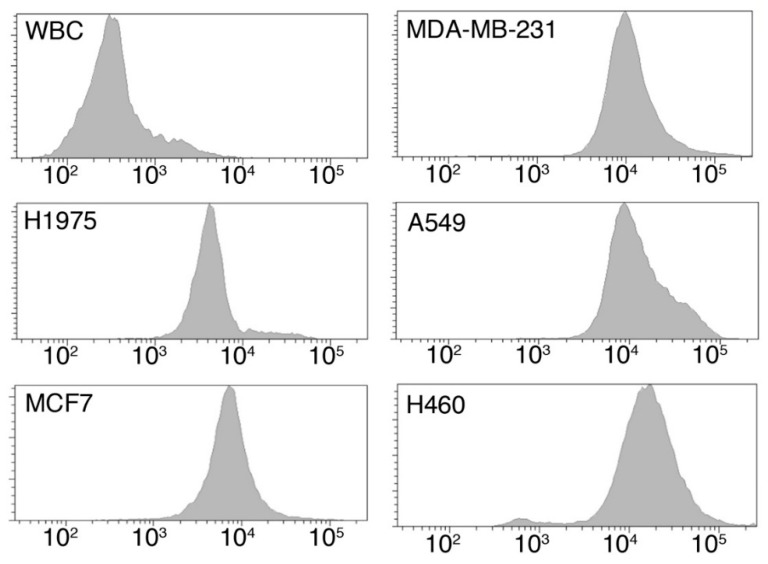
Measurement of glucose uptake by flow cytometry. Representative histograms reporting the 2-NBDG fluorescence of WBCs and of the tested cell lines H1975, MDA-MB-231, MCF7, A549, and H460.

To assess whether tumor cells are characterized by an increased metabolism, with respect to white blood cells (WBC), glucose uptake was evaluates by employing a fluorescent glucose analogue, the 2-(*N*-(7-Nitrobenz-2-oxa-1,3-diazol-4-yl)Amino)-2-Deossiglucose) (2-NBDG). We measured the glucose uptake of both lung cancer (A549, H1975 and H460) and breast cancer (MCF-7 and MDA-MB231) cell lines, as well as that of WBC. As shown in Figure 1, all cancer cell lines showed comparable values of glucose uptake, which resulted to be, in median, more than 10-fold higher than that of WBCs (Figure 1 and Table 1).

**Figure 2:**
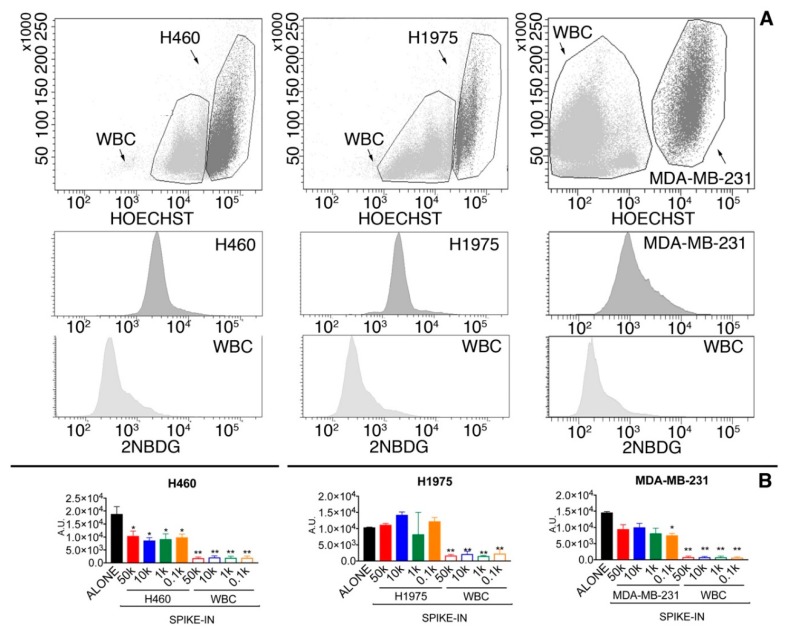
(**A**) Glucose uptake of tumor cells and WBC in spike-in samples. Representative images of spike-in samples (50,000 cells) of H460, H1975, and MDA-MB-231 in WBC. In the upper panels, SSC/Hoechst dot-plots were used to discriminate tumor cells (Hoechst(+), dark grey) from WBC (Hoechst(−), light grey). In the lower panels are presented the histograms showing the 2-NBDG positivity of tumor cells (middle panels) and WBC (lower panels), respectively; (**B**) glucose uptake in tumor cells alone and in spike-in samples. Data are presented as median and interquartile range. Black columns indicate the glucose uptake of tumor cells alone. All the other solid columns refer to different spike-in sample in which 50,000 (50k), 10,000 (10k), 1000 (1k), and 100 (0.1k) tumor cells were spiked into peripheral blood samples. The last four columns indicate the glucose uptake of WBC in the 50k, 10k, 1k, and 0.1k spike-in samples. *, *p* < 0.005 of tumor cells in spike in sample with respect to cells alone (Kruskal–Wallis test followed by Dunn’s post-test). **, *p* < 0.05 of WBC versus the tumor cells of the corresponding spike-in sample (Mann–Whitney test).

To evaluate whether the glucose uptake would remain unchanged if cancer cells were mixed with WBCs, MDA-MB-231 as well as H1975 and H460 were mixed with WBCs (Figure 2). Cancer cells were pre-labeled with Hoechst to make them easily and unequivocally distinguishable from WBCs (Figure 2A). The median glucose uptake of Hoechst(+) cancer cells in spike-in samples was, with respect to cancer cells alone, about 1.5- and 2-fold decreased in MDA-MB-231 and H460 cell lines, while an opposite trend was seen for H1935 cells (Figure 2B). Nonetheless, the distribution curves of the glucose uptake of WBC and Hoechst(+) cancer cells in the spike-in samples were always significantly different (Two-sample Kolmogorov–Smirnov test, *p* < 0.0001), as well as the median intensity of the two populations (Figure 2B).

**Figure 3:**
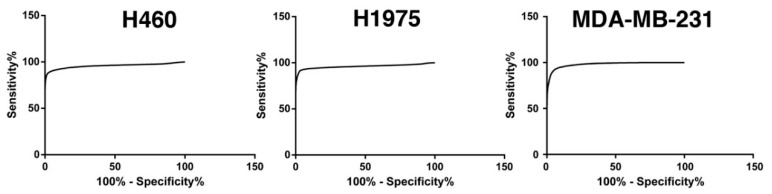
ROC curves obtained analyzing the 2-NBDG positivity of WBC and tumor cells in spike-in samples. See text for more details.

The analysis of the area under the curve (AUC) of the Receiver Operating Characteristic (ROC) curves showed that the glucose-uptake parameter presented an accuracy, in discriminating tumor cells from WBC, of 0.82, 0.96, and 0.96 for MDA-MB-231, H460, and H1975, respectively (Figure 3).

In conclusion, glucose uptake was significantly higher in tumor cell lines with respect to WBC, and this difference remained significant in spike-in samples.

#### 2.1.2. Glucose Uptake Can Be Used to Recover Tumor Cells from Spike-in Samples

To establish the ability of the metabolic assay to recover tumor cells from the peripheral blood, a number from 100 to 10,000 of MDA-MB-231, a consolidated model of EpCAM(−) and metastasis-competent cancer cells, was spiked into peripheral blood. Only cancer cells were pre-labeled with Hoechst to make them easily and unequivocally distinguishable from WBCs. The spiked sample was processed lysing red blood cells and incubating it with the glucose analogue 2-NBDG.

**Figure 4:**
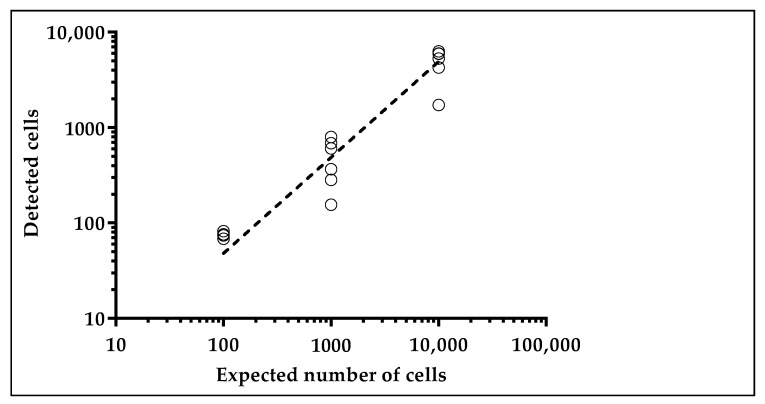
Series of spiking assays with cancer cells pre-labeled with Hoechst. Number of Hoechst positive (detected) cells with respect to the number of spiked ones (expected cells).

The number of Hoechst(+) cancer cells present in the sample after liquid handling was, on average, 55 ± 21% of the spiked ones, with a linear correlation analysis yielding an R-squared of 0.84 (Figure 4). This number is in line with that obtained by other authors and our group using living cells in spike-in experiments and it can be explained not only by the loss of cells due to the handling procedures, but also by the death of part of the spiked cells by anoikis and immuno-mediated phenomena [33,34,35,36].

To create a consistent gating mask to apply to patient samples in order to recognize the highly metabolically active cells, we chose to test operator-independent thresholds based on the 2-NBDG distribution in the WBC population. Specifically, we set the threshold using, as cut-off levels, 3-, 5-, and 7-fold the average intensity of WBCs for 2-NBDG, as well as the 2-NBDG average intensity + 2.5 standard deviation. Table 2 and Supplementary Table S1, indicates, for each assayed cut-off level, which fraction of Hoechst(+) cells was indeed recovered and how many contaminating WBC were present. Decreasing the stringency of the selection, the fraction of recovered CTC increased (from 32.1% to 74.9%), as well as the number of contaminating WBC (from 1412 to 9341). Since the “dilution” of CTC could not exceed the sensitivity of the ddPCR in detecting specific mutations (10^−4^), we set as cut-off level the average uptake of WBC plus 2.5-fold the standard deviation (named threshold “2.5 SD”).

**Figure 5:**
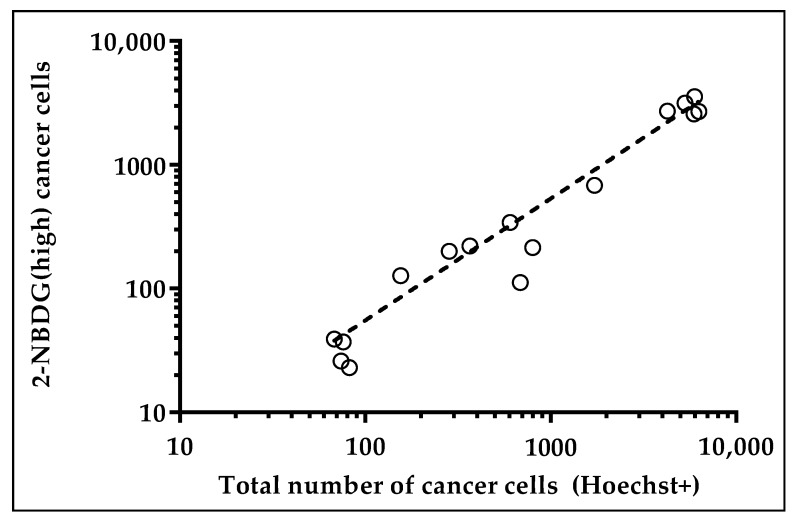
Cancer cells positive for 2-NBDG(+) plotted against the total number of cancer cells (Hoechst(+)) using as the cut-off level “2.5 SD”.

Using as the cut-off level the gate “2.5 SD”, the fraction of 2-NBDG(high) cancer cells displayed a good linearity with the total number of cancer cells, being R^2^ = 0.95 (Figure 5).

#### 2.1.3. Highly Metabolic Cells Sorted from Spike-in Samples by FACS Are Suitable for ddPCR Analysis

**Figure 6:**
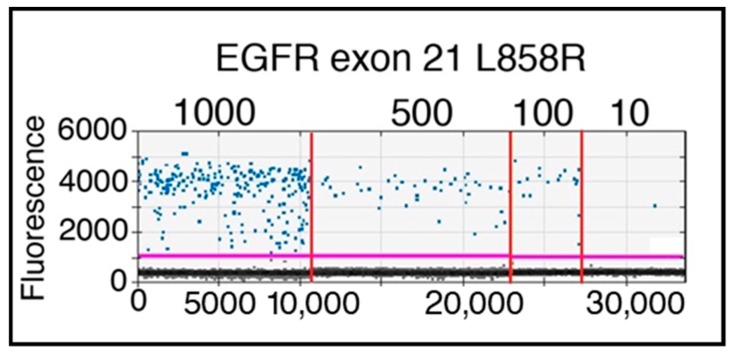
1-D plot showing ddPCR results of spike-in samples performed with H1975 mutated cell line in healthy donor blood sample. The ordinate scale indicates fluorescence intensity. The magenta line is the fluorescence-threshold, above which droplets (blue) containing EGFR p.L858R mutated copies are present. Serial dilutions, from 1000 to 10 H1975 cells, are separated by the vertical red lines.

To establish whether the designed CTC enrichment strategy, based on sorting putative CTC on the basis of their metabolism, would allow us to detect by ddPCR specific gene mutations, 1000, 500, 100, and 10 H1975 cells, harboring the EGFR p.L858R mutation, were spiked in 1 mL of peripheral blood. The presence of the mutation was indeed demonstrated in all the samples sorted adopting the gate “2.5 SD” (Figure 6). As expected on the basis of the recovery rate of living tumor cells from spike-in samples, the number of mutated copies was, on average, 52.2% of the expected ones, although with variable efficiencies (55.4%, 39.2%, 84%, and 38% for 1000, 500, 100, and 10 cells, respectively).

### 2.2. Harvesting and Molecular Analysis of Hypermetabolic Fraction in NSCLC Patients

**Figure 7:**
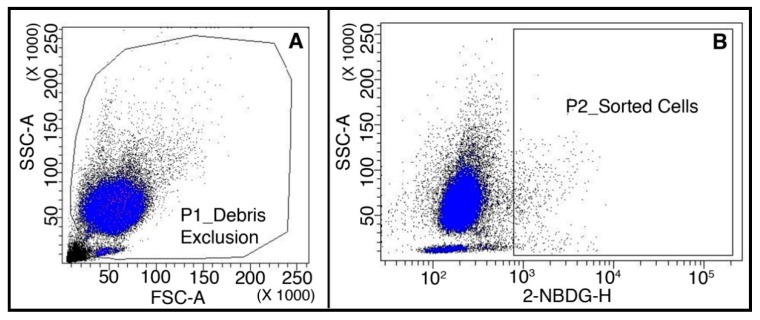
Analysis of a representative patient blood sample. (**A**) Dot-plot showing the morphological parameters Forward- and Side-Scatters; P1 gate selects cells and discards debris; (**B**) dot-plot showing the glucose uptake of P1-gated cells; P2 gate selects cells with the highest intensity of 2-NBDG fluorescence according to the “2.5 SD” threshold.

Samples from patients (*n* = 30) affected by metastatic non-small cell lung cancer were stored at room temperature, and processed and analyzed within 6 h. The storage at room temperature did not affect the cell viability, which was measured on a set of healthy donor samples (*n* = 10) after 3 and 6 h, resulting in 94% ± 4 and 95% ± 3, respectively. A depletion of 90.2 ± 4.8% of CD45(+) cells was achieved in the pre-analytical step using immune-magnetic beads and columns (*n* = 6). Cells presenting the highest uptake of glucose were sorted according to “2.5 SD” threshold (Figure 7). We sorted an average of 4475 ± 4678 cells per sample (range 100–17,000).

**Figure 8:**
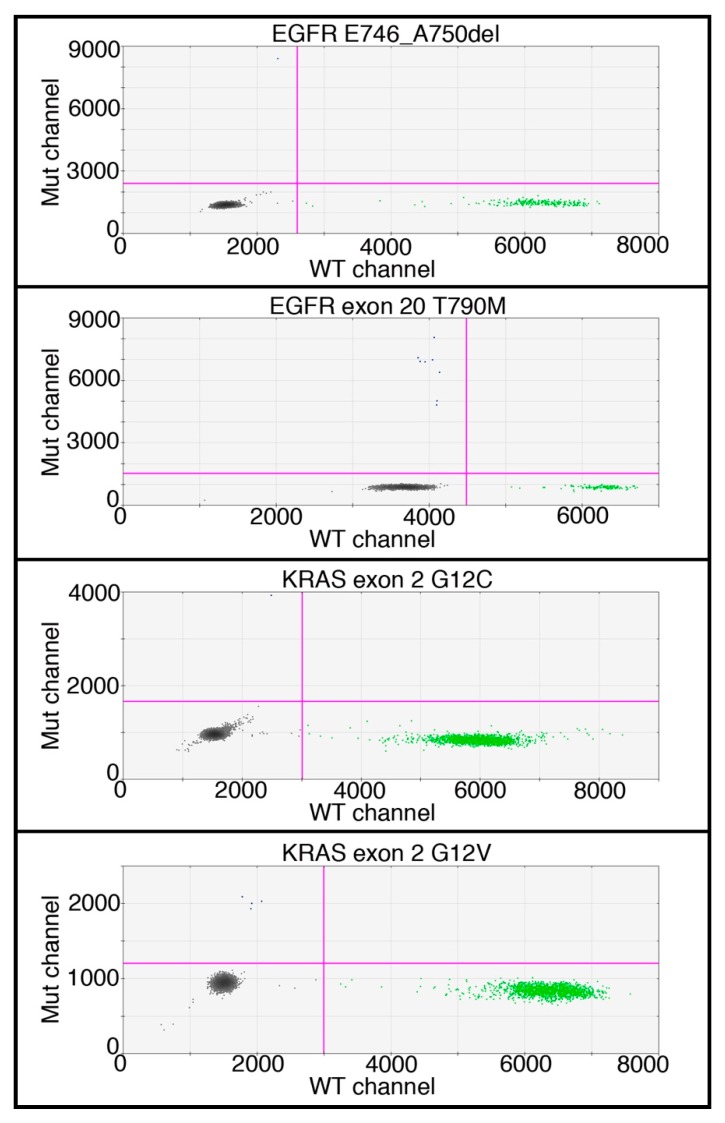
2-dimensional dot-plots showing the results of ddPCR of four representative patients in which mutated DNA was detected. In *Y*-axis: intensity of FAM dye tagging mutated DNA (Mut channel); in *X*-axis: intensity of HEX dye tagging wild type DNA (WT channel). Grey droplets are empty, green droplets contain wild type DNA, and blue droplets contain mutated DNA.

DNA was extracted from the sorted fraction and ddPCR (Figure 8 and Supplementary Table S2) was adopted, to detect the same mutations found in the primary tumor (with three exceptions, described below).

Mutant DNA copies were found in 85% of patients with known mutations on primary tumor (22/26) and in 50% of cases of patients with a wild type primary tumor (2/4) (Table 3).

Considering the cases in which the primary tumor and the 2-NBDG(high) fraction were tested for the same mutations (*n* = 23), an exact matching was found in 70% of the cases (16/23) (Table 3). In an additional case (4.3%), the matching was partial, since the 2-NBDG(high) fraction showed only one of the two mutations of the primary tumor. In two cases (8.7%), the 2-NBDG(high) fraction showed different mutations with respect to the primary tumor. Both cases presented the mutation EGFR p.T790M, known to be involved in drug resistance. Only 17% of the 2-NBDG(high) fraction (4/23) did not show any mutation.

Noteworthy, in the three cases in which it was not possible to evaluate the perfect matching of the mutations of primary tumors and isolated cells, because of the lack of ddPCR probes (patient 4 and patient 27) or an incomplete description of the mutation of the primary tumor (patient 17), the 2-NBDG(high) fraction resulted to harbor mutations on the same gene evaluated on the primary tumors (patient 4 and patient 17) or at least the same mutation that could be analyzed both in the primary tumor and in the sorted cells (patient 27) (Table 3).

As a whole, a new mutation was detected in the 2-NBDG(high) fraction of 8% of the patients with a mutated tumor.

Interestingly, of the four patients with a wild type primary tumor, two showed no mutations, while, among the others, one showed EGFR p.E746_A750del, and the other the KRAS p.G12V mutation. Patients with wild type primary tumor were tested for all mutations, except for the EGFR p.T790M one, because this latter is usually induced by EGFR-inhibitors, not used in these patients.

In order to exclude that the ddPCR could detect false positive events, we tested both the CD45(+) fraction retained in the depletion column (*n* = 17), and a comparable number of cells of the 2-NBDG(low) fraction (*n* = 12); in both fractions we targeted the same mutation tested in the 2-NBDG(high) fraction. Additionally, we evaluated five healthy donor samples for five different mutations, and genomic control DNA were used in each reaction. None of CD45(+) fraction, 2-NBDG(low) fraction, and donor samples were positive for the presence of tested mutations.

In conclusion, the 2-NBDG(high) fraction is enriched in tumor cells whose genetic landscape can be analyzed by ddPCR.

## 3. Discussion

This paper is essentially a proof of principle that, using a metabolic assay coupled with a flow-cytometric analysis, it is possible to sort, at least from NSCLC metastatic patients, CTC that can be genetically analyzed by applying ddPCR, an extremely sensitive technique with a limit of detection of 10^−4^ [37]. In the present form, therefore, our approach is essentially a simple, quick, and inexpensive strategy that can be utilized in every flow-cytometry facility to enrich sample in CTC taking advantage of their metabolism.

Elevated glucose uptake is a well-established cancer feature exploited in positron-emission tomography. The work of other independent groups, in addition to ours, suggests that the same principle can be exploited for CTC detection [21,22,23]. The work of Tang et al. confirms the validity of the concept investigating NSCLC pleural effusions, and showing the detection of hypermetabolic CTC in a limited number of peripheral blood samples [22]. Our work was more focused on peripheral blood and strengthened the evidence collected by Tang et al., showing assessment of EGFR and KRAS mutations in 26 patients. At the same time, the implementation in flow cytometry allows higher standardization and wider diffusion, since currently most institutions have access to a flow cytometry facility. Other approaches, requiring customized microfabricated devices, or dedicated and expensive equipment, have the downside of restricting the availability to specialized labs.

We used ddPCR to confirm that the sorted fraction contained cells originating from the tumor. We observed the presence of multiple mutations in two patients (patient 17 and 26), and the presence of mutations different from the ones detected in the primary tumor in four patients (patients 4, 18, 22, and 24). This suggests that the method can probe intra-patient CTC heterogeneity [38,39], which might be important in tracking disease evolution in serial biopsies.

In this proof of concept, we did not enumerate CTC, choosing to pre-amplify target genes in order to prioritize sensitivity of their detection, though preventing in this way accurate quantitation. CTC enumeration would be theoretically possible by determining both absolute mutant copies and mutant allele frequency by ddPCR, without previous targeted amplification.

One of the main advantages of CTC over ctDNA is the possibility to characterize the “omics” of a single viable tumor cell, or generate CTC-based cell lines for studying mechanisms of metastasis. Metastasis is the main mechanism ultimately causing cancer-related death [40], but it is still poorly understood. Enriched CTC, being the actual cells responsible for metastasis formation in vivo, are excellent candidates for such studies. For both aims, it is important that cells do not undergo severe toxicity during the detection process. The fluorescent metabolite used in the present study is a deoxyglucose analog that is not metabolized, potentially depleting intracellular glucose available to cells [41]. Although no short-term toxicity has been described [42], growth-inhibition of cancer cells by NBDG has been reported through increased oxidative stress, interference with *N*-linked glycosylation, and induction of autophagy [43]. For these reasons, we think that the feasibility of culturing the isolated cells for studies, such as clonal expansion, long-term vitality, and drug sensitivity assays, might not be excluded but needs further validation. Some authors indicate that cell culture of isolated CTC might also suffer of shear stress and high voltage pulse during the sorting procedure [44], although culturing of FACS-sorted cells is an established practice in cell biology.

This method is not limited to CTC with epithelial phenotype, or with increased size, like most of methods presented so far. Furthermore, the method focuses on viable, metabolically active cells, while the other methods often include dead or apoptotic CTC, which might not indicate progression of the disease, as such cells are no longer able to metastasize efficiently.

The presented method could be improved by the addition of CD45 labeling and a double gating on CD45(−), 2-NBDG(high) cancer cells. The antibody labeling on one hand would add cost and complexity to the procedure, but, on the other hand, it would decrease the number of contaminants, potentially enabling sorting of single CTC, which could then be analyzed individually.

Although promising, this is a preliminary study that requires to be validated in an independent case study including a larger number of lung cancer patients. The results can be further strengthened by including both metastatic and non-metastatic patients, since these latter are known to harbor a low number of CTC [12]. Additionally, since in the 2-NBDG(high) fraction of some patients were described mutations not detected in the primary tumor, it would be extremely interesting to add metastasis samples in the mutational analysis. Because metastases are rarely available (this was also our case), the analysis of circulating tumor DNA could represent an alternative.

In conclusion, the presented method has the potential to impact on CTC research field, being a tool for low-cost liquid biopsy with a simple and reproducible procedure.

## 4. Materials and Methods

### 4.1. 2-NBDG Uptake by White Blood Cells and Cancer Cell Lines

To exclude that storage at room temperature could affect blood cells, the viability of WBC from 10 healthy donor samples was assessed, after 3 and 6 h of storage at room temperature, by ADAM-MC automatic cell counter (NanoEnTek, Waltham, MA, USA), according to the manufacturer’s protocol.

To evaluate the 2-NBDG (CAS number 186689-07-6; ThermoFisher, Waltham, MA, USA) uptake, white blood cells (WBCs) were obtained from the peripheral blood of healthy donors (*n* = 3), after red blood cell lysis by ammonium chloride; experiments were performed in triplicate. Human cell lines H1975, MDA-MB-231, MCF7, A549, and H460 (ATCC) were grown according to product sheets, using reagents provided by ThermoFisher (media and antibiotics), and EuroClone (Pero (MI), Italy) (fetal bovine serum). Cells were maintained in culture until confluence, and then detached with 1% trypsin-EDTA (Ethylene-diamine-tetra-acetic acid). The authenticity of the cell lines was assessed by Short Tandem Repeat (STR) DNA profiling (Supplementary Table S3).

WBC and cell lines were incubated separately at 37 °C for 20 min with 350 μM 2-NBDG, washed twice and resuspended in 100 μL of phosphate-buffered saline (PBS) for flow cytometry analysis (FACS Canto II, Becton Dickinson, San Jose, CA, USA). Experiments were performed in triplicate.

### 4.2. Spike-in Assay

Peripheral blood from healthy donors (*n* = 10) was obtained from the Transfusion Medicine of the Academic Hospital of Udine. In order to measure the recovery rate and the accuracy of the method designed for CTC enrichment, known numbers of MDA-MB-231 in a range of 100 to 10,000 were spiked into 250 μL of peripheral blood, while H460, H1975, and A549 were spiked-in in the same range number in 1 mL of peripheral blood to simulate the frequency of rare events. In a set of experiments aimed at evaluating the ability of ddPCR to detect the specific mutation EGFR exon 21 L858R, 10, 100, 500, and 1000 H1975 cells were spiked into 1 mL of peripheral blood. Tumor cells were labeled with the fluorescent nuclear marker HOECHST 33342 (ThermoFisher) and counted using a Burker’s chamber before being spiked into blood donor samples. After red blood cells lysis, 350 µM 2-NBDG [2-(*N*-(7-Nitrobenz-2-oxa-1,3-diazol-4-yl)Amino)-2-Deossiglucose; CAS number 186689-07-6] (ThermoFisher) was added to the sample, which was then incubated at 37 °C for 20 min. Cells were washed twice and resuspended in 100 μL of phosphate-buffered saline (PBS) for flow cytometry analysis.

### 4.3. Patient Samples, Healthy Donors and Clinical Data

The local ethics committee approved the study with protocol number 18387. Written informed consents were obtained from patients and blood donors and all clinical investigations have been conducted according to the principles expressed in the Declaration of Helsinki. Sample collection started February 2016. This feasibility trial was conducted within the Institute of Pathology and the Department of Oncology of the Academic Hospital of Udine.

A total of 28 healthy donors were used for this study: *n* = 3 for 2-NBDG uptake experiments; *n* = 10 to evaluate cell viability upon storage at room temperature; *n* = 5 for the detection of five gene mutations by ddPCR and, finally, *n* = 10 for spike-in samples (see specific sections for details).

For each patient enrolled (*n* = 30), 3 mL of peripheral whole blood were collected into EDTA tubes (BD Vacutainer^®^; BD, Franklin Lakes, NJ, USA). An identification number was assigned to anonymize the samples. All data have been treated according to law and Helsinki criteria.

Inclusion criteria were: 18 years of age or older, diagnosis of NSCLC, Eastern Cooperative Oncology Group (ECOG) performance status between 0–2 without organ dysfunction, presence of metastatic disease confirmed by imaging or pathological analysis, with no discrimination between local (*n* = 13) and distant metastatic sites (*n* = 17). All samples were stored at room temperature and processed within 6 h from the sample collection to minimize the metabolic variability of cells. The clinico-pathological characteristics of the patients included in the study are summarized in Table 4.

KRAS and EGFR mutational status on primary tumor was determined by PCR and quantified by Mass Spectrometry (Myriapod^®^ kit; Diatech Pharmacogenetics, Jesi (AN), Italy) or Real-time PCR (Easy^®^EGFR kit; Diatech Pharmacogenetics) CE-IVD approved assays, according to manufacturer’s instructions. The limit of detection of the tests, according to manufacturers, are 5% and 0.5%, respectively.

### 4.4. Patient Sample Preparation and Staining

Red blood cells were lysed using an ammonium chloride-based lysing reagent (BD Pharm Lyse™; BD Biosciences, San Jose, CA, USA) according to manufacturer’s protocol. Blood samples were depleted of CD45 cells using magnetic micro-beads and LS columns (Miltenyi Biotec, Bergisch Gladbach, Germany), if appropriate (WBC count >5000 cells per µL). Depletion efficiency was assessed by counting a sample before and after depletion with Bürker chamber, in triplicate. Depleted samples were then labeled with 2-NBDG as in spiking assay. For each patient the CD45(+) fraction recovered from the column was used as wild type control of the molecular analysis.

### 4.5. Flow Cytometry and Cell Sorting

Spike-in assays were evaluated using the BD FACS Canto™ (BD Biosciences) and FACS Aria III™ (BD Biosciences).

Patient samples were evaluated using the FACS Aria III™ operated by the FACS DIVA software (BD Biosciences).

The sorting threshold, to define the 2-NBDG(high) population was designed to be operator-independent, standardized, and including at most few thousands of cells, to avoid an excess of contaminants. The threshold was set as follows: data of the first 20,000 blood cells (excluding fragments, and mostly comprising white blood cells) were acquired to assess average intensity (AVGi) of 2-NBDG and standard deviation (SD). Then, the 2-NBDG(high) population was selected as follows:

2-NBDG(high) if 2-NBDG > AVGi + 2.5 × SD

In other words, we sorted out the cells with the highest uptake of glucose, exceeding the average white blood cell of 2.5-fold the standard deviation.

Additionally, a comparable number of 2-NBDG(low) cells was also sorted and further analyzed in 12 patients. This was meant both to prove that the 2-NBDG-dependent sorting was a necessary step to enrich CTC and that the mutated DNA was actually coming from sorted cells, not from cfDNA present in suspension. In fact, cfDNA, if any molecule was left after all washing steps, should have been homogeneously dispersed in the liquid phase, so mutated DNA should have been founded both in the 2-NBDG(high) and the 2-NBDG(low) fraction. If, instead, mutated DNA was actually coming only from hypermetabolic cells, it should have been present only in the 2-NBDG(high) fraction.

### 4.6. DNA Mutation Detection

Genomic DNA isolated from sorted cells after a proteinase K digestion was investigated via ddPCR to prove neoplastic genotype. For patient samples, targeted DNA was pre-amplified using specific primers for EGFR and KRAS (Biorad, Hercules, CA, USA); a single preamplification PCR reaction of 15 cycles was performed for each sample. Although preamplification is not mandatory for ddPCR analysis, considering the variability of the disease, rarity of CTC, and that an estimate number of CTC was not possible for the single patient a priori, for this proof of concept we opted for a light preamplification of target genes, choosing sensitivity in detecting targeted genes over accurate quantification, which was not possible after preamplification. Instead, for spiking assay, where quantification of sorted cells was necessary to evaluate the entire workflow, the preamplification step was by-passed.

Before measuring the mutation levels of targeted genes in patient samples, the preamplification product was diluted 50 times with ddH_2_O. The ddPCR mixture was prepared according to manufacturer’s instructions using the Biorad ddPCR Supermix for Probes (No dUTP) and the specific hybridization probes, conjugated with the dye FAM, for the detection of EGFR p.T790M, p.L858R, and p.E746_A750del and for revealing KRAS mutations p.G12C and p.G12V. The wild type probes both for EGFR and for KRAS were labeled with the dye HEX (ddPCR assay kits, Biorad). All probes were obtained from Biorad, with the following IDs:

EGFR: dHsaCP000039 p.E746_A750del   WT: dHsaCP2000040
EGFR: dHsaCP000019 p.T790M       WT: dHsaCP2000020
EGFR: dHsaCP000021 p.L858R        WT: dHsaCP2000022
KRAS: dHsaCP000007 p.G12C         WT: dHsaCP2500585
KRAS: dHsaCP2500592 p.G12V       WT: dHsaCP2500593

PCR reagents with the DNA extracted from samples (final volume of 20 μL) and 70 μL of oil were loaded into the droplet generator in an 8-well cartridge, in order to generate monodispersed droplets. Then, the emulsion was transferred into a 96-well plate and amplified with a specific thermal profile: 95 °C for 10 min, 40 cycles of 94 °C for 30 s, and 56 °C for 60 s, and a final step at 98 °C for 10 min. After the amplification protocol, the plate was loaded into the QX100 droplet reader (BioRad).

For each ddPCR reaction a wild type control, a mutant positive control and a no-template-control (no DNA) were set up for the correct determination of the fluorescence threshold. The presence of targeted mutations in patient samples were identified and analyzed using the QuantaSoft software (BioRad).

### 4.7. Statistical Analysis

Data are presented as mean ± standard deviation or median and interquartile range, as appropriate. Comparison of medians between two groups were performed by Mann–Whitney tests, while Kruskall–Wallis test followed by Dunn’s post-test was used to compare more than two groups. To compare cumulative distributions, the nonparametric two sample Kolmogorov–Smirnov test was used. *p* < 0.05 was considered significant. Statistical analyses were performed by GraphPad Prism 6 software (GraphPad software, La Jolla, CA, USA).

## 5. Conclusions

In this proof of concept study we demonstrate that CTC can be enriched from NSCLC metastatic patients exploiting their increased glucose uptake via flow cytometry. CTC can then be successfully screened for genetic mutations using ddPCR, revealing both matching and new mutations with respect to the primary tumor analysis. The strength of this method lies in its accessibility to every flow cytometry facility, the relative simplicity of the workflow and the low cost of reagents required. Furthermore, the method is not limited to CTC with epithelial phenotype or increased size, and focuses on viable, metabolically active cancer cells.

## Figures and Tables

**Table 1:** Uptake of glucose analogue by different cell types. The 2-NBDG uptake is expressed as mean fluorescence intensity and standard deviation (SD). Each cell line and WBC from three different healthy subjects were tested in triplicate. * *p* value obtained comparing cell lines and WBC by Mann–Whitney test.

Cell Type	Origin	Median Fluorescence Intensity (A.U.)	*p*-Value *
WBC	Healthy donor	291	-
H1975	Lung Cancer	4758	0.0238
MDA-MB-231	Breast Cancer	8338	0.0043
MCF-7	Breast Cancer	6983	0.0238
A549	Lung Cancer	9977	0.0238
H460	Lung Cancer	19,764	0.0095

**Table 2:** Performances of the metabolic assay depending on the selected threshold. The table shows, for each assayed cut-off level used to define cells as 2-NBDG(high), tumor cell recovery, and number of WBC contaminants. Tumor cell recovery was defined as the number of 2-NBDG(high) Hoechst(+) cancer cells/total number of Hoechst(+) cancer cells × 100. WBC contaminants were defined as the number of white blood cells present in the 2-NBDG(high) population. Cut-off levels were defined as 3-, 5-, and 7-fold the average intensity of the WBC for 2-NBDG as well the average 2-NBDG fluorescence intensity + 2.5-fold its standard deviation (gate “2.5 SD”). Data are presented as average values between all the analyzed spike-in samples.

Cut-Off Level	Tumor Cell Recovery	WBC Contaminants
3-fold WBC average	74.9%	9341
5-fold WBC average	46.5%	3052
7-fold WBC average	32.1%	1412
WBC average + 2.5-folds SD	59.8%	4473

**Table 3:** Comparison between the mutations found in the primary tumor and in the 2-NBDG(high) sorted fraction. In bold, we highlighted the mutations detected in the cells that were different or additional with respect to those found in the primary tumor.

	Primary Tumor	2-NBDG(high)	Matching
PATIENT 1	KRAS p.G12C	KRAS p.G12C	Perfect
PATIENT 2	KRAS p.G12C	KRAS p.G12C	Perfect
PATIENT 3	KRAS p.G12C	KRAS p.G12C	Perfect
PATIENT 4 *	KRAS p.G12D	**KRAS p.G12C**	n.a.
PATIENT 5	EGFR p.L858R	EGFR p.L858R	Perfect
PATIENT 6	EGFR p.E746_A750del and EGFR p.T790M	EGFR p.E746_A750del and EGFR p.T790M	Perfect
PATIENT 7	EGFR p.E746_A750del	EGFR p.E746_A750del	Perfect
PATIENT 8	EGFR p.L858R	EGFR p.L858R	Perfect
PATIENT 9	EGFR p.L858R and EGFR p.T790M	EGFR p.Leu858Arg and EGFR p.T790M	Perfect
PATIENT 10	EGFR p.L858R	EGFR p.L858R	Perfect
PATIENT 11	EGFR p.E746_A750del	EGFR p.E746_A750del	Perfect
PATIENT 12	EGFR p.E746_A750del	EGFR p.E746_A750del	Perfect
PATIENT 13	EGFR p.L858R	EGFR p.L858R	Perfect
PATIENT 14	EGFR p.E746_A750del	EGFR p.E746_A750del	Perfect
PATIENT 15	EGFR p.E746_A750del	EGFR p.E746_A750del	Perfect
PATIENT 16	EGFR p.E746_A750del	EGFR p.E746_A750del	Perfect
PATIENT 17 **	KRAS exon 2 [not specified]	KRAS p.G12C and KRAS p.G12V	n.a.
PATIENT 18	EGFR p.E746_A750del	**EGFR p.T790M**	Different
PATIENT 19	KRAS p.G12C	WT	NEG
PATIENT 20	KRAS p.G12C	WT	NEG
PATIENT 23	EGFR p.L858R and EGFR p.T790M	EGFR p.T790M	Partial
PATIENT 25	EGFR p.E746_A750del	**EGFR p.T790M**	Different
PATIENT 26 **	KRAS p.G12C	KRAS p.G12C and **KRAS G12V**	Additional
PATIENT 27 ***	EGFR p.G719C and EGFR p.T790M	EGFR p.T790M	n.a.
PATIENT 29	KRAS p.G12V	WT	Negative
PATIENT 30	KRAS p.G12C	WT	Negative
PATIENT 21	WT	WT	Perfect
PATIENT 22	WT	**EGFR p.E746_A750del**	New mutation
PATIENT 24	WT	**KRAS p.G12V**	New mutation
PATIENT 28	WT	WT	Perfect

* Patient 4—Like for the other patients, KRAS p.G12C and p.G12V mutations, but not p.G12D, were tested.t. ** Patient 17—The mutational status of the primary tumor was not available in full detail: only the mutated oncogene, without additional details, was present in the records. *** Patient 27—we tested for the EGFR p.T790M mutation, we did not test for EGFR p.G719C. n.a.: not applicable, either due to unavailability of detailed information on primary tumor mutations or unavailability of specific probes.

**Table 4:** Demographic, clinical, and pathological characteristics of the 30 patients enrolled.

**Characteristic**	**Years, mean (range)**
Age	68 (51–87)
**Characteristic**	**Number of patients (% on total)**
**Gender (*n* of patients (%))**	
Male	16 (53.3%)
Female	14 (46.7%)
**Histological subtype (*n* of patients (%))**	
Adenocarcinoma (ADC)	29 (96.7%)
Unknown	1 (3.3%)
**Mutation (*n* of patients (%))**	
Mutation of the primary tumor:	26 (86.7%)
EGFR	16 (61.5%)
p.E746_A750del	9 (34.6%)
p.L858R	6 (23.1%)
p.T790M	4 (15.4%)
KRAS	10 (38.5%)
p.G12C	8 (30.8%)
p.G12V	1 (3.8%)
Unknown	1 (3.8%)
Wild-type primary tumor	4 (13.3%)
**ECOG Performance Status (*n* of patients (%))**	
0	18 (60%)
1	9 (30%)
2	2 (6.7%)
Unknown	1 (3.3%)
**Stage (*n* of patients (%))**	
IV	29 (96.7%)
Unknown	1 (3.3%)
**Metastatic status (*n* of patients (%))**	
Mx	1 (3.3%)
M1	29 (96.7%)
**Metastatic sites (*n* of patients (%))**	
Lung	21 (70%)
Pleural effusion	11 (36.7%)
Lymph nodes	3 (10%)
Bone	10 (33.3%)
CNS	6 (20%)
Adrenal gland	2 (6.7%)
Liver	3 (10%)
Kidney	1 (3.3%)

## Supplementary Materials

The following are available online at http://www.mdpi.com/2072-6694/10/8/270/s1, Table S1: Raw data of spike-in experiments of MDA-MB-231 cells into blood samples; Table S2: Raw data of the ddPCR assays of the 2-NBDG(high) cells, isolated from 30 patients affected by stage IV NSCLC; Table S3: STR analyses of cancer cell lines.
