# Supplementary material for: Assessment of the Mutational Status of NSCLC Using Hypermetabolic Circulating Tumor Cells
Source: Cancers (Basel). 2018 Aug 14;10(8):270. doi: 10.3390/cancers10080270 (PMC6115779; doi:10.3390/cancers10080270)
Supplement: Supplementary file 1 [file cancers-10-00270-s001.pdf]

cancers MDPI

Supplementary Table S1. Raw data of spike-in experiments of MDA-MB-231 cells into blood samples. The Table shows how the threshold, used to define the 2-NBDG(high) population, affects both the number of the recovered tumor cells (Hoechst(+)/2-NBDG(+)) and the number of contaminating WBC (Hoechst(-)/2-NBDG(+)). Data are presented as mean ± standard deviation of 4 different experiments. 4 different thresholds have been tested.

Threshold 1 for 2-NBDG(high) cells: 3-fold the average intensity of WBC for the 2-NBDG.

Spiked Cells	Hoechst(+) cells (tumor cells in the spiked samples)	Hoechst(+)/2-NBDG(high) (tumor cells present in the 2-NBDG(high) fraction)	Hoechst(-)/2-NBDG(high) (contaminating WBC present in the 2-NBDG(high) fraction)
50,000	23,186 ± 1558	21,164 ± 2035	9657 ± 3113
10,000	5397 ± 829	4064 ± 926	6896 ± 2496
1000	595 ± 136	456 ± 109	5437 ± 1454
100	73 ± 6	52 ± 9	1005 ± 501

Threshold 2 for 2-NBDG(high) cells: 5-fold the average intensity of WBC for the 2-NBDG.

Spiked Cells	Hoechst(+) cells (tumor cells in the spiked samples)	Hoechst(+) 2-NBDG(high) (tumor cells present in the 2-NBDG(high) fraction)	Hoechst(-)/2-NBDG(high) (contaminating WBC present in the 2-NBDG(high) fraction)
50,000	23,186 ± 1558	13,849 ± 2703	5553 ± 1748
10,000	5397 ± 829	2500 ± 557	3676 ± 1826
1000	595 ± 136	220 ± 156	2909 ± 932
100	73 ± 6	25 ± 3	480 ± 212

Threshold 3 for 2-NBDG(high) cells: 7-fold the average intensity of WBC for the 2-NBDG.

Spiked Cells	Hoechst (+) cells (tumor cells in the spiked samples)	Hoechst(+) 2-NBDG(high) (tumor cells present in the 2-NBDG(high) fraction)	Hoechst(-)/2-NBDG(high) (contaminating WBC present in the 2-NBDG(high) fraction)
50,000	23,186 ± 1558	8726 ± 3516	1983 ± 937
10,000	5397 ± 829	1467 ± 703	1235 ± 274
1000	595 ± 136	113 ± 68	1209 ± 618
100	73 ± 6	14 ± 4	201 ± 501

Threshold 4 for 2-NBDG(high) cells: average intensity of WBC + 2.5 fold the standard deviation.

Spiked Cells	Hoechst(+) cells (tumor cells in the spiked samples)	Hoechst(+) 2-NBDG(high) (tumor cells present in the 2-NBDG(high) fraction)	Hoechst(-)/2-NBDG(high) (contaminating WBC present in the 2-NBDG(high) fraction)
50,000	23,186 ± 1558	20,097 ± 2361	9010 ± 3398
10,000	5397 ± 829	3921 ± 1010	4071 ± 503
1000	595 ± 136	428 ± 94	3434 ± 1342
100	73 ± 6	47 ± 11	745 ± 490

cancers MDPI

Supplementary Table S2. Raw data of the ddPCR assays of the 2-NBDG(high) cells, isolated from 30 patients affected by stage IV NSCLC.

	Mutation found by ddPCR	Total Number of droplets per assay	Number of Mutated droplets	Number of Wild Type droplets
PATIENT 1	KRAS p.G12C	10,000	1	9524
PATIENT 2	KRAS p.G12C	10,000	48	9830
PATIENT 3	KRAS p.G12C	9000	25	7916
PATIENT 4	KRAS p.G12C	8000	13	6026
PATIENT 5	EGFR p.L858R	9000	15	6726
PATIENT 6	EGFR p.T790M	7000	132	6686
PATIENT 7	EGFR p.E746_A750del	5000	54	3571
PATIENT 8	EGFR p.L858R	7000	7	6428
PATIENT 9	EGFR p.T790M	5000	11	4572
PATIENT 10	EGFR p.L858R	14,000	415	7939
PATIENT 11	EGFR p.E746_A750del	5000	42	94
PATIENT 12	EGFR p.L858R	6000	116	2343
PATIENT 13	EGFR p.E746_A750del	3000	2	2268
PATIENT 14	EGFR p.E746_A750del	7000	282	5975
PATIENT 15	EGFR p.E746_A750del	7000	274	6207
PATIENT 16	EGFR p.E746_A750del	4000	1	1021
PATIENT 17	KRAS p.G12C	8000	1	6522
PATIENT 17	KRAS p.G12V	6000	5	4690
PATIENT 19	KRAS p.G12C	13,000	0	808
PATIENT 21	EGFR p.E746_A750del	14,000	0	216
PATIENT 22	EGFR p.E746_A750del	10,000	7	6110
PATIENT 23	EGFR p.T790M	6000	5	5382
PATIENT 24	KRAS p.G12V	11,000	3	1731
PATIENT 25	EGFR p.T790M	12,000	5	4852
PATIENT 26	KRAS p.G12V	14,000	5	11,959
PATIENT 26	KRAS p.G12C	13,000	1	11,409
PATIENT 27	EGFR p.T790M	10,000	3	2539

Supplementary Table S3. STR analyses of cancer cell lines.

Cell line	STR profile ATCC	STR profile extraction 21/02/18
**MCF7**	Amelogenin: X	Amelogenin: X
	CSF1PO: 10	CSF1PO: 10
	D13S317: 11	D13S317: 11
	D16S539: 11, 12	D16S539: 11, 12
	D5S818: 11, 12	D5S818: 11, 12
	D7S820: 8, 9	D7S820: 8, 9
	THO1: 6	THO1: 6
	TPOX: 9, 12	TPOX: 9, 12
	vWA: 14, 15	vWA: 14, 15
		D21S11: 29, 30

Cell line	STR profile ATCC	STR profile extraction 30/03/2018
**A549**	Amelogenin: X, Y	Amelogenin: X, Y
	CSF1PO: 10, 12	CSF1PO: 10, 12
	D13S317: 11	D13S317: 11
	D16S539: 11, 12	D16S539: 11, 12
	D5S818: 11	D5S818: 11
	D7S820: 8, 11	D7S820: 8, 11
	THO1: 8, 9.3	THO1: 8, 9.3
	TPOX: 8, 11	TPOX: 8, 11
	vWA: 14	vWA: 14
		D21S11: 29

Cell line	STR profile ATCC	STR profile extraction 09/07/2018
**H460**	Amelogenin: X, Y	Amelogenin: X, Y
	CSF1PO: 11, 12	CSF1PO: 11, 12
	D13S317: 13	D13S317: 13
	D16S539: 9	D16S539: 9
	D5S818: 9, 10	D5S818: 9, 10
	D7S820: 9, 12	D7S820: 9, 12
	THO1: 9.3	THO1: 9.3
	TPOX: 8	TPOX: 8
	vWA: 17	vWA: 17
		D21S11: 30
